# State-of-the-Art Techniques to Causally Link Neural Plasticity to Functional Recovery in Experimental Stroke Research

**DOI:** 10.1155/2018/3846593

**Published:** 2018-05-27

**Authors:** Anna-Sophia Wahl

**Affiliations:** ^1^Brain Research Institute, University of Zurich, Winterthurerstr. 190, 8057 Zurich, Switzerland; ^2^Department of Health Sciences and Technology, ETH Zurich, Winterthurerstr. 190, 8057 Zurich, Switzerland; ^3^Central Institute of Mental Health, University of Heidelberg, J5, 68159 Mannheim, Germany

## Abstract

Current experimental stroke research faces the same challenge as neuroscience: to transform correlative findings in causative ones. Research of recent years has shown the tremendous potential of the central nervous system to react to noxious stimuli such as a stroke: Increased plastic changes leading to reorganization in form of neuronal rewiring, neurogenesis, and synaptogenesis, accompanied by transcriptional and translational turnover in the affected cells, have been described both clinically and in experimental stroke research. However, only minor attempts have been made to connect distinct plastic remodeling processes as causative features for specific behavioral phenotypes. Here, we review current state-of the art techniques for the examination of cortical reorganization and for the manipulation of neuronal circuits as well as techniques which combine anatomical changes with molecular profiling. We provide the principles of the techniques together with studies in experimental stroke research which have already applied the described methodology. The tools discussed are useful to close the loop from our understanding of stroke pathology to the behavioral outcome and may allow discovering new targets for therapeutic approaches. The here presented methods open up new possibilities to assess the efficiency of rehabilitative strategies by understanding their external influence for intrinsic repair mechanisms on a neurobiological basis.

## 1. Introduction

Although huge efforts have been made in recent years, both by clinicians and basic researchers, we have still gained limited insights into a neurological disease such as stroke preventing us from developing specific cures and resulting in poor statistical numbers: Of 15 million people suffering from an ischemic brain attack every year, a third dies, a third remains permanently disabled, and a third recovers as the stroke itself has not been too devastating. On the clinical side, stroke units have been created, which combine experts in intensive care medicine, neurology, physiotherapy, and speech therapy, to accelerate and coordinate the diagnostic and therapeutical processes aiming at improving recovery rates for patients. According to the neurologists' saying “time is brain,” even mobile units have been established to bring the hospital to the patient [1]. These efforts aim at increasing the number of patients being eligible for the only currently approved acute treatment—thrombolysis or thrombectomy—within a very early time window of 4.5 h after stroke [2, 3].

On the side of basic research, we study the neurobiology of stroke, but seem to be stuck in a “black box” situation: We have accumulated data showing the tremendous capacities of the brain to reorganize by synaptogenesis and even neurogenesis and by neuronal circuit rewiring and new circuit formation. We find cortical map shifts and hyperactive brain regions after stroke; we detect genetic and proteomic turnover within a distinct spatiotemporal profile and sequence of events [4]. However, only minor attempts have been made to transform pure correlative data into causative ones, which would enable a causal connection of plastic remodeling processes in the brain with distinct behavioral outcomes. Not only would this allow us to form a new understanding of the functional brain status after stroke, but also it opens up possibilities to develop and test the efficiency of new therapeutic approaches. Today's basic stroke research is part of neuroscience that faces the challenges to first describe the broad morphological features, then study fine cellular and molecular events, find genes which are active in a specific neuron or cell type, and link it to the behavioral phenotype. But as the philosopher of science Karl Popper might have argued: Before we can provide answers, we need the power to ask new questions.

In recent years, new technology has been designed which is starting to fill the gap between correlative and causative research.

The aim of this review is to first discuss current state-of-the-art technology of experimental stroke research which enables a deeper understanding of neuronal reorganization and circuit formation. In the second part, techniques for distinct neuronal circuit manipulation are introduced which help to reveal causal relationships between anatomy and behavior. Finally, new approaches combining cytology with molecular profiles are provided which elucidate the underlying molecular mechanisms of neuronal rewiring and repair. The principles of the techniques are explained together with exemplary studies in experimental stroke research which have already applied the described methodology. The here discussed tools may not only enhance our understanding of stroke pathology but also help to identify crucial anchor points for new therapeutic interventions.

## 2. State-of-the-Art Techniques to Study Neuronal Reorganization and Circuit Formation after Experimental Stroke

### 2.1. Approaches to Examine Brain-Wide Remodeling

A classical approach to study reorganizational processes across the whole brain is functional resonance imaging (fMRI), allowing the monitoring of neuronal rewiring processes on a macroanatomical level within the same animal. However, although significant contributions to the understanding of the interplay between altered functional status and structural connectivity have been made in stroke models [5, 6] using this technique, the spatial and temporal resolution level remains low.

In contrast, intrinsic optical imaging sticks out by a high spatial resolution enabling the visualization of small domains within larger brain areas demonstrating the functional organization and the spatial relationships among those smaller domains, for example, in the barrel or visual cortex [7]. Intrinsic optical imaging uses the effect that more active brain tissue reflects less light than does less active tissue. Thus, the most active areas appear as the darkest ones. Optical imaging has been used to show disrupted functional connectivity in rodent mouse models of stroke [8, 9].

Another technique to study in particular sensory map shifts is millisecond-timescale voltage-sensitive dye (VSD) imaging which unlike functional fMRI and intrinsic optical signal imaging measures electrical activity with relatively high spatial and temporal resolution [10]. VSD imaging has recently been applied to measure spontaneous activity over large regions of the mouse cortex to reveal fast, complex, localized, and bilaterally synchronized patterns of depolarization [10]. In a study by Gosh et al. [11], VSD imaging was used to show the expansion of the forelimb sensory map towards parts of the hindlimb cortex after a large thoracic spinal cord injury, indicating incorporation of axotomized hindlimb neurons into sensory circuits of the forelimb. In another study by Brown et al. [12], the function of the sensorimotor cortex was visualized with VSD imaging. The mouse forelimb sensory cortex was targeted by stroke leading to a new sensory representation in the territory previously occupied by the forelimb motor cortex. VSD imaging revealed slower kinetics in remapped sensory circuits accompanied by high levels of dendritic spines as visualized with two-photon microscopy.

While VSD imaging was the first demonstration of wide-field optical imaging of neural activity [13], the necessity to apply VSDs prior to imaging, their very fast response time, and their very small signal ratios made them difficult to use. However, recent developments of exogenous and in particular genetically encoded fluorescent indicators of neuronal activity such as GCaMP and YC-Nano have revolutionized the targeted expression of fluorescence with much higher signal levels. And even transgenetic lines exist [14]. Furthermore, recent work has shown that the dynamics of flavoprotein fluorescence can be optically mapped in wild-type CNS tissue as an indicator of oxidative metabolism [15, 16], which might be also a useful tool in experimental stroke research. As wide-field neuroimaging methods are also relatively easily combined with optogenetics to modify brain activity in the behaving animal, studies have been conducted using camera-based “mesoscopic” optical recording of neuronal activity examining a large part of the cortical surface. These mesoscopic optical recordings became possible by extensive chronic window implantations [17] and improved high-speed sensitive camera technology. Vanni et al. [18] measured cortical functional connectivity using wide-field imaging in lightly anesthetized GCaMP3 mice and correlated calcium signals recoded in the primary sensory cortex to the other sensorimotor areas bilaterally. The coactivation of areas was interpreted as an indication that areas might be functionally connected. However, a final proof for a functional connection, shutting-off correlated areas, was missing in this study. Balbi et al. [19] used the same approach in awake GCaMP6 mice studying mososcopic functional connectivity longitudinally in a microinfarct model.

That wide-field calcium imaging might be also a powerful tool to study brain-wide cortical reorganization on a high-resolution level over time in animals monitoring their rehabilitative courses or recording rehabilitative training after stroke was demonstrated by Murphy et al. [20]. His lab presented a home cage system, where mice could initiate mesoscopic functional imaging by themselves over days being unsupervised.

### 2.2. Methods to Understand Regional Reorganization

Intracortical microstimulation (ICMS) and surface stimulation with electrode arrays have been used for many years to map cortical regions, to study cortical reorganization, and to find first hints if projections in the motor cortex are functionally relevant. This technique applies electrical stimulation of cortical sites to induce, for example, stimulus-evoked movement responses, which can be detected visually, by EMG responses or by the usage of accelerometers. Several studies have used this technique to either examine cortical map shifts in sensorimotor areas after spontaneous recovery or different therapeutical applications [21–24] or used the stimulation itself as a method to increase plastic processes [25]. However, ICMS has its disadvantages such as the inability to selectively target neuronal subtypes as well as the indiscriminate activation of axons of passage. Furthermore, due to electrode penetration, intracortical electric stimulation remains an invasive procedure causing tissue damage [26]. ICMS is also limited to perform cortical representation of body function at a distinct time point after stroke constraining longitudinal experiments within the same animal.

A new noninvasive strategy to study the reorganization of the motor cortex after stroke in the same animal over time is light-based motor mapping: This technique makes usage of the possibility to stimulate neurons by light, either by uncaging neurotransmitters [27, 28] or by directly activating light-sensitive channels, such as channelrhodopsin-2 (ChR-2). Ayling et al. [26] used transgenic channelrhodopsin-2 mice which express ChR-2 in layer 5B pyramidal neurons of the motor cortex. Thus, light-based stimulation directly targets corticofugal cells, enabling the analysis of their contribution to motor cortex topography. Light-based motor mapping has the advantage of sampling stimulus-evoked movements at hundreds of cortical locations in mere minutes objectively and in a reproducible manner [29]. It is faster and less invasive than electrode-based mapping and can be combined with intrinsic signal imaging in animals with cranial window preparations [30]. In addition, it enables repeated mapping of the motor cortex over a timescale of minutes to months, opening up possibilities to examine the dynamics of movement representations at distinct conditions such as learning over time, pharmacological intervention or reorganization before, during, and after cortical damage. In a first study by Harrison et al. [29] light-based motor mapping revealed a functional subdivision of the forelimb motor cortex based on the direction of movements evoked by brief light pulses (10 ms), while prolonged stimulation (100–500 ms) resulted in complex movements of the forelimb to specific positions in space. In a follow-up study [30], light-based mapping was for the first time used to perform a longitudinal experiment studying the reorganization of the sensorimotor cortex after a focal sensory stroke. The sensory stroke caused the establishment of a new sensory map in prior parts of the forelimb motor cortex, which preserved its center position but became more dispersed.

### 2.3. Studying Poststroke Neuronal Circuit Formation and Network Activity

However, although all described mapping approaches are powerful tools, they can only provide information about map shifts and representation of general movement dynamics. They stay far beyond cellular resolution and do not allow studying local neuronal circuitry or single neuron contribution to neuronal networks. In particular after stroke, it is not clear how activity in single neurons changes in relation to cortical map shifts. The analysis of single neurons in relation to the neuronal circuit in which they are embedded may elucidate whether stroke-induced plasticity is a result of the capacity of surviving neurons to process multiple functional streams. In vivo two-photon calcium imaging is a potent method which not only allows studying activity of a single neuron or ensembles of neurons in a network but also enables cell type and neuronal subtype-specific analysis. Only a few in vivo two-photon calcium imaging studies focusing on neuronal reorganization and circuit rewiring after stroke have been conducted so far. In an acute in vivo calcium imaging experiment during the induction of a transient global ischemia model in mice, Murphy et al. [31] saw a widespread loss of mouse somatosensory cortex apical dendritic structure during the phase of ischemic depolarization. This was accompanied by increased intracellular calcium levels which coincidently occurred with the loss of dendritic structure. In a second study [32], in vivo two-photon calcium imaging was used to examine how response properties of individual neurons and glial cells in reorganized forelimb and hindlimb functional somatosensory maps modified during the recovery period from ischemic damage in the sensory cortex. However, all studies have been conducted in animals under anesthesia which itself influences neuronal activity. An experiment which examines single neuron activity in the behaving animal before stroke and during the recovery phase after insult is lacking so far.

Two-photon calcium imaging enables recordings of individual neuronal activity within a neuronal network and allows subtype-specific functional analysis of brain tissue. However, neuronal activity with cellular resolution level can only be examined in small (<1 millimeter) fields of view. Collective dynamics across different brain regions are inaccessible. Recent advances in two-photon microscopy allow the simultaneous imaging of neuronal networks with cellular resolution level in the active animal in multiple areas, which are even not directly connected [33–35]. This technological progress also provides new promises for experimental stroke research.

## 3. State-of-the-Art Techniques for Manipulating Neuronal Circuits

In a 1979 Scientific American article, Nobel laureate Francis Crick stated that the major challenge facing neuroscience was the need to control one type of cell in the brain while leaving others unaltered. In a lecture from 1999, he further confined: “One of the next requirements is to be able to turn the firing of one or more types of neuron on and off in the alert animal in a rapid manner. The ideal signal would be light, probably at an infrared wavelength to allow the light to penetrate far enough. This seems rather farfetched but it is conceivable that molecular biologists could engineer a particular cell type to be sensitive to light in this way” [36].

Manipulation of neuronal circuits or single neurons has two prerequisites: Manipulation has to be quick and very specific. Over the years, a very diverse set of tools has been developed to manipulate whole brain regions as well as the activity of individual cells and subtypes in the alert behaving animal.

### 3.1. Manipulating with Specific Spatial Control

The first constraint for specific manipulation implies a high spatial control allowing the selective modulation of a whole brain area, a distinct anatomical sub region (e.g., layer 5 pyramidal cells in the sensorimotor cortex) or a particular cell type (e.g., a parvalbumin-positive interneuron).

For inhibiting neuronal activity in whole brain regions, agents such as the GABA agonist muscimol have been used [37, 38] resulting in a loss of motor function, indicating a causal relationship between anatomy and behavior. Other approaches block synapse remodeling through protein synthesis inhibitors such as anisomycin, for example, inducing the disruption of synapses and motor maps in a rat forelimb stroke model [39]. However, for a better spatial control to target distinct circuits and individual cell types, researchers have either created transgenic mouse lines or locally injected viruses with cell-type specific promotors [40]. These promotors induce gene expression directly—as in the case of transgenic mice—or indirectly via, for example, Tet- (tetracycline-controlled transcriptional activation-) on/off or Cre-flox systems.

The Tet system uses at least two viral vectors plus an antibiotic drug which in a sequential way activate each other to induce the transcription and translation of the gene of interest: A tissue-specific promoter initiates the expression of a transcription factor, either the tetracycline transactivator (tTA) or the reverse tetracycline transactivator (rtTA). The tTA or rtTA then becomes the key player for the transcription of a tetracycline response element (TRE) promotor, which in dependence of the presence of tetracycline or doxycycline drives the expression of the gene of interest. Expression of the gene of interest is fully reversible as administration or removal of tetracycline or doxycycline will turn its expression on or off. In a study by Kinoshita et al. [41] a Tet-on system was used to selectively express the synaptotoxin tetanus toxin in propriospinal (PN) neurons innervated by the motor cortex. The researchers could show that upon doxycycline administration in the drinking water reaching performance of monkeys significantly declined due to temporal blockade of the motor cortex–PN–motor neuron pathway. The same Tet-on system was used by Wahl et al. [42] in a rat stroke model to selectively shut off rewired corticospinal fibers originating in the contralesional pre- and motor cortex and targeting the stroke-denervated hemi-spinal cord: When doxycycline was administered to the rats in the drinking water after the rehabilitative treatment for impaired skilled motor function, the recovered grasping skills of the impaired paw decreased again over time. The effect was reversible, when doxycycline was removed from the drinking water. This study showed for the first time the specific and reversible inactivating of newly out-sprouting corticospinal fibers after stroke. In another study by Ishida et al. [43] ipsilesional corticorubral fibers were shut off after forced rehabilitation in a rat stroke model revealing the functional importance of the red nucleus for the recovery of impaired motor function.

Similar to the Tet system, the Cre-flox system also requires two transgenes: A tissue-specific promotor regulates the expression of Cre recombinase, a bacteriophage enzyme which recombines DNA at specific recognition sequences called loxP sites. Cre recombinase then excises DNA within two loxP sites (“floxed”). As in most cases, floxed-stop constructs are knocked in by homologous recombination to a gene of interest [40]; the stop signal is excised in the presence of Cre and the transgene expression can be initiated. Cre-mediated expression only occurs in cells expressing Cre, which are also those cells in which the tissue-specific promotor is active, indicating the high cell-type specificity of this technique.

### 3.2. Manipulating with High Spatial and Temporal Control

#### 3.2.1. DREADDs

As the second prerequisite for specific neuronal manipulation in addition to a high degree of spatial resolution, temporal resolution and directional modulation of signaling are required for remotely controlling neuronal firing. Temporal resolution implies the precise control when a receptor or pathway is active or inactive and for how long it should be in a specific active status. Temporal resolution can vary from milliseconds (see “opsins” described below) to hours (e.g., designer receptors exclusively activated by designer drugs, DREADDs). Important are also “onset” kinetics (the time between the experimental manipulation and the modulation of the receptor or signaling pathway) and “offset” kinetics (the time between the initiation of the signaling modulation and the termination of the modulation [40]). Directional regulation describes the effect of the tool on neuronal activity (either activating or inhibiting), while bidirectional control would be the optimal case: Turning on and off the same cell population would elucidate the full spectrum of function that a cell provides within a particular network for perception or execution of a distinct behavior.

For manipulation of neuronal networks for minutes to hours, designer G protein-coupled receptors have been developed. G protein-coupled receptor pathways are involved in a multitude of cellular functions. Unlike opsins, which are functionally silent without excitation in vivo as they are not directly activated by endogenous compounds, G protein-coupled receptors (GCPRs) are constantly modulated by endogenous ligands in vivo or reveal ligand-independent activity [44, 45]. In vitro and in vivo pharmacological studies have described GPCRs as the most important class of druggable targets in the human genome [46], through which 50% of prescribed therapeutics act [47]. These facts made the development of highly selective orthologous ligand-receptor pairs, which would enable a high spatiotemporal control over GPCR signaling pathways in vivo challenging [48]. In recent years, mutations to more than a dozen native GPCRs have opened the field for the development of selectively activated designer receptors. Most of these receptors are divided in two classes: the first-generation RASSLs (receptors activated solely by synthetic ligands) and the second-generation DREADDs (designer receptors exclusively activated by designer drugs), which were evolved through directed molecular evolution in yeast [40]. RASSLs were first engineered on the basis of serotonin receptors [49], histamine receptors [50], and melanocortin-4 receptors [51]. However, the first generation of orthologous ligand-GPCR pairs revealed potential shortcomings: Although the receptors were activated solely by the synthetic ligands, the ligands themselves did not solely activate the designer receptors (as reviewed by Rogan and Roth [40]). Thus, for the development of second-generation DREADDs, Armbruster et al. took a designer ligand, clozapine n-oxide (CNO), which was known to be inert at endogenous targets and highly bioavailable and blood-brain barrier-permeant in both humans and mice [52, 53]. As CNO had a modified structure of clozapine, which is known to be a weak partial agonist at muscarinic receptors, [54] mutations were induced in the five members of the muscarinic cholinergic receptor family and tested upon their selective responsiveness to the CNO application. Introducing two mutations transformed the hM3 receptor into a designer receptor which was insensitive to its native ligands, but highly sensitive to the designer ligand CNO. In smooth muscles cells the G_q_-coupled hM3 DREADD receptor stimulated a cascade of inositol phosphate hydrolysis, calcium release and ERK1/2 activation, while the hM4Di DREADD receptor, derived from the G_i_-coupled human muscarinic M4 receptor, inhibited forskolin-induced cAMP formation and activation of GIRK causing hyperpolarization and inhibition of neuronal firing [54].

Since the first development of DREADD receptors, reports of their usage in vivo are now appearing: The pharmacokinetic properties of the DREADD ligands and the particular route of administration (oral administration, subcutaneous, intraperitoneal, or even local stereotaxic infusion) determine how quickly neurons response to experimental manipulation by ligand application. Responses typically emerge 5 to 15 min after systematic application, for example, of CNO and usually last for 2 h—but this time period can be further enlarged upon dose-dependent increase of CNO [55]. When Ferguson et al. [56] used virus-mediated expression of the hM4Di receptor in the direct and indirect pathway neurons of the striatum, they found altered behavioral plasticity associated with repeated drug treatment. In particular, decreasing striatopallidal neuronal activity facilitated behavioral sensitization to drug treatment. Expression of the hM4Di receptor in rewired corticospinal projecting neurons was recently used to shut off regained grasping function in rats which had gained nearly full recovery of impaired skilled forelimb function due to a large stroke after the sequential application of a growth-promoting therapy and intense rehabilitative training [42].

#### 3.2.2. Optogenetics

Although manipulation of GPCR signaling pathways by DREADD receptor induction and activation is highly efficient and shows a very specific spatial resolution (depending on the constructs or transgenic mouse lines used), the temporal resolution remains limited to an activation within minutes—due to the slower nature of GPCR signaling—and the necessary ligand delivery to the location of neuronal manipulation. In contrast, high-temporal (milliseconds) and cellular precision within intact mammalian neural tissue for fast, specific excitation or inhibition even within a freely moving animal can only be achieved with optogenetics [57].

Early approaches to use light to stimulate neuron activity included the selective photostimulation of neurons in *Drosophila* by coexpression of the drosophila photoreceptor genes encoding arrestin-2, rhodopsin, and the alpha subunit of the cognate heterotrimeric G protein which enabled the sensitization of neurons to light [58]. In a second approach, action potentials in hippocampal neurons were induced in a reliable and temporarily precise manner by uncaging a caged capsaicin derivate by light [59]. However, depolarization occurred within 5 s after a 1 s light pulse, lasting for 2–3 s and did not attenuate with multiple light pulses. Other approaches such as UV light-isomerizable chemicals linked to genetically encoded channels [60, 61] had also shown limitations due to reduced speed, targeting, tissue penetration, or applicability because of their multicomponent nature [57]. In 2003, Nagel et al. [62] cloned channelrhodopsin-2 (ChR2), a cation channel from the green alga *Chlamydomonas reinhardtii* which depicted similarities to the vertebrate rhodopsin which opens in response to blue light allowing potassium ions to enter the cell. Two years later, the first optogenetic experiment in neuroscience was conducted by expressing ChR2 using a lentiviral vector in cultured rat hippocampal neurons [63]. Illumination of these cultures with shorter wavelength blue light (450–490 nm) initiated large and rapid depolarization, while light with longer wavelengths (490–510 nm) induced smaller currents. Light stimulation of neurons was selective to those neurons expressing ChR2. Since then, neuroscientists rapidly adapted the possibilities of this new technology to in vivo experiments. In addition, the palette of available light-sensitive channels and ion pumps for neuronal inhibition and activation, for fast- and slow-acting opsins, and for opsins activated at distinct wavelengths has been extensively augmented in recent years [64–68].

However, although the numerous advantages of optogenetics are evident—such as the highest specificity, the ultrafast millisecond time scale dissection, and basically no adverse effects due to the light (unless the light source is not too strong or applied too long)—optogenetics stays an invasive procedure for many in vivo experiments: As the light source has to be brought close to the neuronal tissue, targeting deep brain areas or diffuse neuronal populations remains challenging. New development of step function or bistable opsins and opsins such as Jaws—an inhibitory opsin, which is activated by light of infrared wavelength [66]—opens up new possibilities for noninvasive manipulation in-vivo.

Only very few studies have applied optogenetics in experimental stroke research so far: Optogenetics was mainly used in the context of light-based motor mapping as described above [30]. Other studies used optogenetics as a therapeutic approach to increase neuronal activity aiming at enhancing functional recovery: In a first study by Cheng et al. [69], optogenetic stimulation of the ipsilateral primary motor cortex in ChR2 transgenic mice promoted functional recovery and the induction of growth-promoting genes after stroke induction in the striatum and somatosensory cortex. Shah et al. [70] could furthermore show that selectively stimulating neurons in the lateral cerebellar nucleus (LCN), a deep cerebellar nucleus that sends major excitatory output to multiple motor and sensory areas in the forebrain, results in persistent recovery on the rotating beam after stroke in a transgenic mouse line (Thy1-ChR2-YFP-channelrhodopsin fused to yellow fluorescent protein under the Thy1 pan-neuronal promoter). Tennant et al. [71] revealed that optogenetic stimulation of thalamocortical axons could facilitate recovery. In another study [72], optogenetic stimulation of the intact corticospinal tract was sufficient to promote functional recovery after a large photothrombotic stroke in rats. Optogenetics were also used to drive the excitatory outputs of the grafted neural stem cells and increase forelimb use on the stroke-affected side and motor activity in a rat stroke model [73]. Reducing the inhibitory striatal output by optogenetics enhanced neurogenesis in the subventribular zone and behavioral recovery in mice after middle cerebral artery occlusion [74–76].

That optogenetics can also be used to demonstrate a causal relationship between a rewiring neuronal circuit and recovery of a specific (sensorimotor) behavior was demonstrated by Wahl et al. [72]: The authors used the inhibitory light-sensitive proton pump ArchT to reveal the functional relevance and regionalized organization of rewired corticospinal circuitry for the recovery of distinct grasping features.

New advanced technology in microscopy allows the precise optogenetic stimulation of individual neurons [77] and even dendritic spines and nerve cell somata [78, 79] using holographic photostimulation. In addition, the development of parallel illumination methods [80] which combine the preservation of the spatial targeting capability of beam-scanning systems and the rapid stimulation of multiple neurons now enable the simultaneous excitation of neurons in selected target regions.

#### 3.2.3. Magnetogenetics

Although optogenetics have revolutionized the field of neuroscience, the examination of deeper, subcortical brain regions remains a challenge, as the light has to be somehow delivered to the tissue often requiring invasive implantation of fiber optics causing collateral damage of the surrounding brain tissue. A new emerging method which overcomes the spatial limitations is magnetogenetics: It relies on a principle known as thermal relaxation [81], implying that an alternating magnetic field is able to heat up small magnetic nanoparticles. As key elements the specific frequency of the magnetic field, the size and composition of the nanoparticles are required. Huang et al. [82] activated a heat-sensitive TRPV1 channel expressed in human embryonic kidney (HEK) cells by induction of thermal relaxation of manganese oxide nanoparticles, which enhanced the temperature at the plasma membrane and initiated the calcium influx through the heat-sensitive ion channels. Chen et al. [83] used this technique to stimulate a defined neuronal population activity in the ventral tegmental area in behaving mice demonstrating the potential of magnetogenetics for deep brain stimulation.

However, although individual neuron and specific neuronal circuit manipulation with high spatial and temporal control is possible as discussed above, there still is a need for good behavioral readouts: In particular, in experimental stroke research studying, for example, motor impairment and recovery, it is crucial to quantitatively understand true recovery versus compensation of impaired function [84]: While analyzing video recordings of motor behavior using scores is not only time consuming but also often very subjective, even the analysis of movement trajectories might not provide the full picture [72]: When manipulating with high precision control on a cellular and even subcellular level on the neurobiological side, there is a need for precise analysis of the behavioral phenotype. The dramatic development of Computer Vision algorithms and artificial intelligence may allow further steps beyond for a detailed analysis of kinematics including the sequence of postures, shape and trajectories, which is missed by the human eye.

## 4. State-of-the-Art Techniques to Combine Anatomy and Molecular Biology

So far, we have discussed how stroke reorganization can be examined on the macrolevel of map shifts or by studying single neurons in neuronal circuits using 2-photon calcium imaging approaches. We have reviewed how individual neurons and whole neuronal populations can be manipulated with high spatiotemporal resolution disclosing new possibilities of causally linking individual neuronal activity with a distinct behavioral phenotype. However, an understanding of the underlying molecular crosstalk which induces anatomical and behavioral changes is still lacking. Classically, tracing techniques (e.g., dextran tracers) have been applied to visualize cells involved in structural reorganization after stroke [22, 23]. Li et al. [85] found a way to exclusively study molecular changes in newly out-sprouting neurons (“the sprouting transcriptome”) in the peri-infarct cortex by injecting two different fluorescent conjugates of the tracer cholera toxin B (CTB) into forelimb sensorimotor cortex at different times points: One CTB tracer was injected at the time of stroke, the second differently labeled one either 7 or 21 days afterwards. Neurons which expressed only the second tracer were those which missed an axonal projection to the injection site at the time of the injection of the first tracer and thus represented neurons which established a new projection pattern after stroke. Both neuron types (single- and double-labeled ones) were laser captured to identify the distinct transcriptional profile of an out-sprouting neuron in the peri-infarct cortex.

In addition, new constructs have been recently developed for molecular profiling of projecting neurons and thus bridging the gap between anatomical modifications and underlying molecular mechanisms. Using, for example, bacterial artificial chromosome (BAC) transgenic mice which express EGFP-tagged ribosomal protein L10a in defined cell populations allowed purification of polysomal mRNAs from genetically defined cell populations in the brain [86, 87]. In another study by Ekstrand et al. [88], ribosomes were tagged with a camelid nanobody raised against GFP enabling the selective capture of translating mRNAs in projecting neurons.

## 5. Conclusion

Here, we have reviewed current and new promising state-of-the-art techniques for studying reorganization after stroke, for the identification and manipulation of distinct neuronal populations and approaches which allow examining molecular profiles of neurons being part of the cortical reorganization process. While for decades studies in basic stroke research have only described and reported correlative findings, these techniques open up tremendous possibilities to analyze plastic processes and identify and target key players for the development of new therapies in stroke.
